# Platelet function in HIV plus dengue coinfection associates with reduced inflammation and milder dengue illness

**DOI:** 10.1038/s41598-019-43275-7

**Published:** 2019-05-08

**Authors:** Eugenio D. Hottz, Anna Cecíllia Quirino-Teixeira, Rogério Valls-de-Souza, Guy A. Zimmerman, Fernando A. Bozza, Patrícia T. Bozza

**Affiliations:** 1 Laboratório de Imunofarmacologia, Instituto Oswaldo Cruz (IOC) – Fundação Oswaldo Cruz (FIOCRUZ), Rio de Janeiro, Brazil; 2 Laboratório de análise de glicoconjugados, Departamento de Bioquímica, Instituto de Ciências Biológicas (ICB) – Universidade Federal de Juiz de Fora (UFJF), Minas, Gerais Brazil; 3 Laboratório de doenças febrís agudas, Instituto Nacional de Infectologia Evandro Chagas (INI), FIOCRUZ, Rio de Janeiro, Brazil; 4 Molecular Medicine Program, Department of Internal Medicine, University of Utah, Salt Lake City, Utah USA; 5 Laboratório de Medicina Intensiva, INI, FIOCRUZ, Rio de Janeiro, Brazil; 6 Instituto D’Or de Pesquisa e Ensino (IDOr), Rio de Janeiro, Brazil

**Keywords:** Innate immunity, Infectious diseases

## Abstract

HIV-infected subjects under virological control still exhibit a persistent proinflammatory state. Thus, chronic HIV infection changes the host homeostasis towards an adapted immune response that may affect the outcome of coinfections. However, little is known about the impact of HIV infection on inflammatory amplification and clinical presentation in dengue. Platelets have been shown to participate in immune response in dengue and HIV. We hypothesized that altered platelet responses in HIV-infected subjects may contribute to altered inflammatory milieu and disease progression in dengue. We prospectively followed a cohort of 84 DENV-infected patients of whom 29 were coinfected with HIV under virological control. We report that dengue and HIV coinfection progress with reduced inflammation and milder disease progression with lower risk of vascular instability. Even though the degree of thrombocytopenia and platelet activation were similar between dengue-infected and HIV plus dengue-coinfected patients, plasma levels of the platelet-derived chemokines RANTES/CCL5 and PF4/CXCL4 were lower in coinfection. Consistently, platelets from coinfected patients presented defective secretion of the stored-chemokines PF4 and RANTES, but not newly synthesized IL-1β, when cultured *ex vivo*. These data indicate that platelets from HIV-infected subjects release lower levels of chemokines during dengue illness, which may contribute to milder clinical presentation during coinfection.

## Introduction

With the emergence of combined antiretroviral therapy (ART) the epidemiology of human immunodeficiency virus (HIV) infection has changed in the last decades from high mortality by opportunistic infections in acquired immunodeficiency syndrome (AIDS) to long-term noninfectious comorbidities in subjects chronically infected with HIV^1,2^. Even though sustained virologic control is achieved through ART, higher rates of long-term comorbidities including cardiovascular diseases, HIV-associated neurocognitive disorders and non-AIDS cancers are still responsible for increased morbidity among HIV infected people^3–7^. Long-term complications in people living with HIV have been attributed to continuing immune suppression and a persistent pro-inflammatory state that are still observed after years of viral suppression by ART^4,8–12^. In addition to favoring the development of long-term non-infectious comorbidities, this altered inflammatory milieu may affect the outcome of non-opportunistic community-acquired coinfections favoring resistance, tolerance or pathology. Nevertheless, the immune network and key cellular components involved in HIV-driven reprogramming of host homeostasis are highly complex, and little is known about the impact of chronic HIV infection on the pathogenesis and clinical presentation of endemic/epidemic infectious diseases, including dengue.

Dengue is an arthropod-born viral disease caused by one of four antigenically-related dengue viruses (DENV-1 to -4). It is the most frequent hemorrhagic viral disease and re-emergent infection in the world^13,14^. Recently, it was estimated that over 2.5 billion people live in high-risk transmission areas with more than 90 million symptomatic infections occurring annually^14^. DENV infection induces a spectrum of clinical manifestations that range from mild self-limited dengue fever to life-threatening severe dengue. While mild dengue presents as undifferentiated febrile illness, severe dengue syndrome progress with hemodynamic dysfunction including hypovolemia, hypotension, shock, bleeding and eventually organ dysfunction and death^13,15–17^. The pathophysiologic mechanisms underlying severe dengue are not completely understood but an overwhelming immune activation with increased production of proinflammatory cytokines and chemokines is considered to favor DENV pathology and severity^18–22^. Interestingly, DENV infection in people living with HIV under stable ART has been associated with lower rates of severe dengue^23^. Nevertheless, the mechanisms and cellular components involved in milder clinical presentations in dengue patients coinfected with HIV are not known.

Thrombocytopenia is a hallmark of dengue infection and has been also observed in average 20% of HIV-infected subjects in post-ART era^24^. The drop of platelet counts in dengue patients correlates with the level of hemodynamic instability and severity of illness, while its recovery associates with clinical improvement and hospital discharge^16,18,25–28^. Blood platelets, classically known as specialized cells in hemostasis, are getting increasingly recognized as major inflammatory cells with key roles in the innate and adaptive arms of the immune system^29–31^. Recently, the contributions of platelets to inflammatory amplification and disease pathogenesis have been identified in both dengue and HIV^32–41^. Even through platelet activation has been extensively investigated in HIV infected subjects and dengue patients, platelet responses in patients co-infected with dengue and HIV were not previously addressed.

Here we show that patients coinfected with HIV plus dengue progressed with milder clinical presentation compared to patients infected with DENV only. More benign disease progression in coinfected patients was associated with reduced levels of pro-inflammatory cytokines and platelet-derived chemokines. Detailed evaluation of platelet features demonstrated no differences in the degree of thrombocytopenia, platelet apoptosis or platelet activation. However, platelets isolated from HIV plus dengue coinfected patients showed defective secretion of granule-stored chemokines when cultured *ex vivo*. These data suggest that platelet degranulation fatigue with reduced secretion of stored chemokines may contribute to reduced inflammatory response in HIV plus dengue coinfection.

## Material and Methods

### Human Subjects

We prospectively followed a cohort of 84 serologically and/or molecularly confirmed DENV-infected patients examined at the Instituto Nacional de Infectologia Evandro Chagas (INI) – Fundação Oswaldo Cruz, Rio de Janeiro, Brazil, of whom 29 were coinfected with HIV-1. Patients with dengue or HIV plus dengue were followed up to the recovery so data from patients were compared to the same subjects after recovering from dengue illness. Clinical, laboratorial and demographic characteristics of dengue infected and HIV plus dengue coinfected patients are summarized in Tables 1 and 2. All HIV infected subjects were under stable ART and had undetectable viral load at the time of inclusion. Two nucleoside reverse-transcriptase inhibitors (NRTI) plus one non-nucleoside reverse-transcriptase inhibitor (NNRTI) or two NRTI plus one protease inhibitor busted with ritonavir (PI/r) were the most prevalent ART schemes (Table 2). The median CD4+ T cell count before DENV infection was 604 cells/mm^3^ (IQR 479.5–700) and there was no evidence of opportunistic infection in any HIV infected subject. Peripheral vein blood samples were obtained at the febrile (n = 46), critical (n = 49) and recovery (n = 15) phases of DENV infection. The median day of sample collection was 3 (IQR 2–4.8) days after de onset of symptoms for dengue and 4 (IQR 3–4.5) days for HIV + dengue at the febrile phase; 6 (IQR 5–8) days for dengue and 6 (IQR 5–7.8) days for HIV + dengue at the critical phase, and 16 (IQR 12.5–21.5) days for dengue and 14 (12–35) days for HIV + dengue at the recovery. All patients had the clinical charts reviewed up to the recovery and patients were classified as having mild dengue (51.2%), dengue with warning signs (44.0%) or severe dengue (4.8%) according with WHO case definition^13^. Primary and secondary infections were distinguished using the IgM/IgG antibody ratio as previously described^42–44^. Viral RNA was extracted from plasma using the QIAamp Viral RNA mini-kit (Qiagen, CA) and processed for virus typing and quantification as previously described^45,46^. The study protocol was approved by the Institutional Review Board (Instituto Nacional de Infectologia Evandro Chagas #016/2010) and the experiments were performed in compliance with this protocol. Written informed consent was obtained from all included patients prior to any study related procedure in accordance with the Declaration of Helsinki.

**Table 1:** Characteristics of dengue infected and HIV plus dengue co-infected patients.

	Dengue (55)	HIV + Dengue (29)	*p* value
Age, years	35 (28–45)	41 (33–46)	0.3697
Gender, male	31 (56%)	20 (68%)	0.3483
Platelet count, ×10^3^/mm^3^	109 (80.5–153.8)	137.5 (84–169.5)	0.4053
Hematocrit, %	40.2 (37.5–42.3)	38.8 (34.9–40.1)	**0.0241**
Albumin, g/dL	3.6 (3.4–3.9)	3.6 (3.3–4.0)	0.7806
TGO/AST, IU/L	53.5 (31.3–106.8)	51 (33.5–100.8)	0.9678
TGP/ALT, IU/L	74.5 (49–123.8)	57.5 (46–83)	**0.0463**
Mild dengue	26 (47.2%)	17 (58.6%)	0.3647
Dengue with warning signs^a^	26 (47.2%)	11 (37.9%)	0.4911
Severe dengue^b^	3 (5.5%)	1 (3.5%)	1.0000
Hemorrhagic manifestations	24 (43.6%)	8 (27.6%)	0.1656
Major bleed^c^	11 (20%)	1 (3.45%)	**0.0501**
Petechiae and exanthema	18 (32.7%)	5 (17.24%)	0.1979
Intravenous fluid resuscitation	18 (32.7%)	14 (48.3%)	0.2371
Secondary infection^d^	41 (74.5%)	21 (72.4%)	1.0000
PCR positive	27 (49.1%)	15 (51.7%)	0.6494
DENV-1	15 (55.5%)	10 (66.6%)	0.5115
DENV-2	0 (0%)	1 (6.6%)	0.3750
DENV-4	12 (44.5%)	4 (26.6%)	0.3175
IgM positive	48 (87.3%)	24 (82.8%)	0.7440
IgG positive	45 (81.8%)	26 (89.7%)	0.5276
NS1 positive^e^	18 (32.7%)	9 (31%)	1.0000

Data are expressed as median (interquartile range) or number (%). ^a^Abdominal pain or tenderness, persistent vomiting, clinical fluid accumulation, mucosal bleed or increased hematocrit concurrent with rapid decrease in platelet count, according to WHO criteria (2009). ^b^Severe plasma leakage, fluid accumulation and severe bleeding, according to WHO criteria (2009). ^c^Gastrointestinal bleed, vaginal bleed and/or hematuria. ^d^Patients that have been previously infected by a different DENV serotype. ^e^DENV nonstructural protein 1 (NS1) antigenemia. ALT, alanine aminotransferase; AST, aspartate aminotransferase; TGO, glutamicoxalacetic transaminase; TGP, glutamic-pyruvic transaminase.

**Table 2:** Virologic, immunologic and therapeutic characteristics of HIV infected subjects before dengue coinfection.

	HIV infected subjects (29)
CD4+ T cell count/mL	604 (479.5–700)
HIV-1 viral load, copies/mL	Undetectable (<50)
Time since HIV diagnosis, years	10 (6–14)
ART scheme:	
NRTI + NNRTI	12 (41.4%)
NRTI + PI/r	12 (41.4%)
Raltegravir-based	3 (10.3%)
NRTI + NNRTI + PI/r	2 (6.9%)

Data are expressed as median (interquartile range) or number (%). NRTI, nucleoside reverse-transcriptase inhibitors; NNRTI, non-nucleoside reverse-transcriptase inhibitors; PI/r, protease inhibitors busted with ritonavir.

### Platelet Isolation

Platelets were isolated as previously described^33,47^. Briefly, peripheral blood samples were drawn into anticoagulant acid-citrate-dextrose (ACD) and centrifuged at 200 × g for 20 minutes to obtain platelet-rich plasma (PRP). Platelets were precipitated from PRP by centrifugation at 500 × g for 20 min in the presence of 100 nM Prostaglandin E_1_ (PGE_1_) (Cayman Chemicals) to avoid platelet activation. The supernatant was discarded, and the platelet pellet was resuspended in 25 mL of PSG (PIPES-saline-glucose: 5 mM C_8_H_18_N_2_O_6_S_2_, 145 mM NaCl, 4 mM KCl, 50 mM Na_2_HPO_4_, 1 mM MgCl_2_-6H_2_O and 5.5 mM glucose) containing 100 nM of PGE_1_. The platelet suspension was centrifuged at 500 × g for 20 minutes, the supernatant discarded and the pellet resuspended in medium 199 (Lonza) to the concentration of 10^9^ platelets/mL. The purity of the platelet preparations (>99% of CD41^+^) was confirmed by flow cytometry.

### Flow Cytometry analysis

Platelets (1–5 × 10^6^) were incubated with FITC-conjugated anti-CD41 (BD Phamingen) (1:20) and PE-conjugated anti-CD62-P (BD Pharmingen) (1:20) for 30 min at 37 °C. Phosphatidylserine exposure on platelets was measured by the binding of FITC-conjugated Annexin V (BD Pharmingen); mitochondrial membrane potential (Δ_Ψ_m) was measured using the probe tetramethylrhodamine ethyl ester (TMRE, Fluka Analytical) (100 nM, 10 min), active caspase-9 was determined using the probe FAM-LEDH-FMK green fluorescent inhibitor of caspase (FLICA, Immunochemistry Technologies), and platelet synthesis of nitric oxide (NO) was quantified using the probe DAF-FM diacetate (Invitrogen Molecular probes). For intracellular IL-1β labeling, isolated platelets were labeled with FITC-conjugated anti-CD41, fixed with 4% paraformaldehyde for 20 minutes, washed once, and permeabilized with Triton 0.1% for 10 minutes. Platelets were then incubated for 30 minutes with anti-IL-1β antibody (5 mg/mL; Santa Cruz Biotechnology), followed by incubation with secondary Alexa Fluor 546–conjugated anti-rabbit IgG for 30 minutes. Isotype-matched antibodies were used to control nonspecific binding of all antibodies. Platelets were distinguished by the expression of CD41 and characteristic forward and side scattering. A minimum of 10,000 gated events were acquired using a FACScalibur flow cytometer (BD Bioscience, CA).

### Cytokines and Chemokines measurement

Plasma samples were collected from ACD-anticoagulated blood and frozen in liquid nitrogen until use. The levels of the cytokines IL-1β, IL-1Ra, IL-8, IFN-γ, IFN-α, IP-10, MCP-1, RANTES, MIP-1β and TNF-a in plasma were measured using a Multiplex cytokine immunoassay (Bio-Plex Human Cytokine Assay). Levels of PF4/CXCL4 and MIF were determined using a standard capture ELISA Kit (R&D Systems) according to manufacturer’s instructions.

Platelets (10^9^ per mL) isolated from 10 dengue-infected and 6 HIV + dengue coinfected patients were incubated at 37 °C in a 5% CO_2_ atmosphere. After 4 hours of incubation, platelets were pelleted, the supernatants were harvested and the secreted levels of PF-4/CXCL4, RANTES/CCL5 and IL-1β were measured using standard ELISA protocol according to manufacturer’s instructions (R&D systems). PF4/CXCL4, RANTES/CCL5 and IL-1β were also measured in supernatants of platelets obtained from the same patients at the recovery phase.

### Statistical analysis

Statistics was performed using GraphPad Prism, version 7.0 (GraphPad, San Diego, CA). The demographic and clinical variables were expressed as median and interquartile range (25–75 percentile) or as number and percentage (%). All numerical variables were tested for normal distribution using the Kolmogorov-Smirnov test. For comparisons among three groups we used Oneway ANOVA to determine differences and Bonferroni’s multiple comparison test to locate the differences among groups. For comparisons between two groups we compared the continuous variables using the t test for parametric distribution or the Mann Whitney U test for nonparametric distribution. The paired two-tailed t-test was used to compare the levels of cytokines secreted *ex vivo* by platelets from the same patients at the acute and the recovery phase. Qualitative variables were compared using Epi-Info software version 7.0 (CDC) to determine the size of effect by calculating odds ratio (OR) and confidence interval (CI) and by the two-tailed Fisher test to determine the p values.

## Results

### Dengue infection of people living with HIV associates with benign evolution of dengue illness

**Figure 1:**
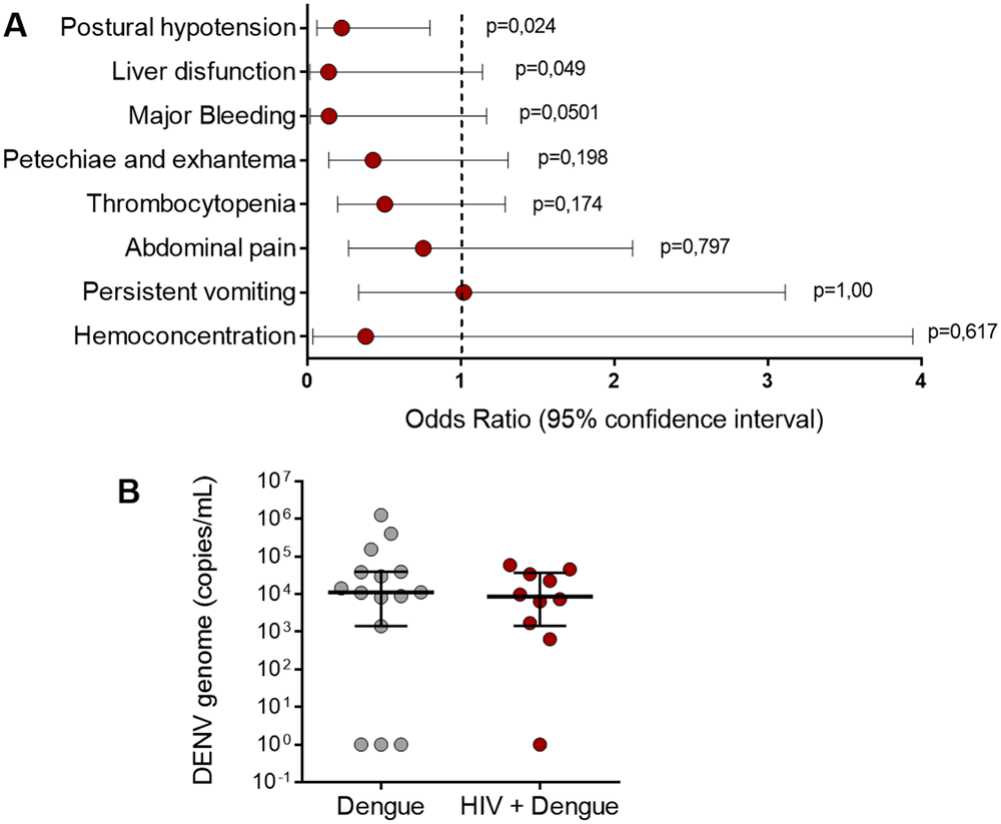
Clinical outcomes and viremia in patients infected with dengue or coinfected with dengue and HIV. (**A**) The odds ratio (OR) of presenting clinical signs of severity in dengue was calculated for each variable in the context of presence or absence of HIV coinfection. The following outcomes were considered: postural hypotension, liver dysfunction (hepatomegaly and/or steatosis evidenced by abdominal ultrasonography plus liver enzymes greater than 200 U/L), major bleeding (Gastrointestinal bleed, vaginal bleed and/or hematuria), petechiae and exanthema, thrombocytopenia (<100,000 platelets/µL), abdominal pain, persistent vomiting and hemoconcentration (increase in hematocrit more than 20% from the value at the recovery). Dots represent the OR and whiskers indicate 95% confidence interval. The dotted line represents OR = 1, in which the exposure confers no protection nor risk. OR values (and 95% CI) above 1 represent increased risk and OR values below 1 represent lower risk of presenting the outcome compared to unexposed (HIV-negative) patients. (**B**) DENV genome copies in plasma from patients with dengue or with HIV + dengue coinfection. Each dot represents the concentration of DENV RNA in plasma from one patient. Horizontal lines indicate the median and interquartile ranges.

Based on the review of the clinical charts from 55 dengue infected and 29 HIV plus dengue coinfected patients, we investigated the relationship between chronic HIV infection (exposure) and severity of dengue illness (outcome) (Fig. 1A and Table 1). Coinfection with HIV has been associated with more benign clinical presentation of dengue illness in a previous publication^23^. In this cohort, HIV plus dengue coinfected patients were significantly protected from postural hypotension (OR [95% CI] = 0.225 [0.063–0.779], p = 0.0245) compared to dengue infected patients without HIV coinfection (Fig. 1A). Moreover, HIV plus dengue coinfected patients presented a trend towards protection against liver dysfunction (hepatomegaly and/or steatosis evidenced by abdominal ultrasonography, plus liver enzymes greater than 200 U/L) (OR [95% CI] = 0.1396 [0.0171–1.1421], p = 0.0491) and major bleeding (Gastrointestinal bleed, vaginal bleed and/or hematuria) (OR [95% CI] = 0.1429 [0.0175–1.1681], p = 0.0501) (Fig. 1A and Table 1). In agreement, hematocrit values and the levels of plasma glutamic-pyruvic transaminase (TGP) were significantly reduced in dengue patients coinfected with HIV when compared to patients infected with dengue virus only (Table 1). Altogether, these data indicate that dengue infection of people living with HIV under stable ART is associated with reduced vascular instability and liver damage.

**Figure 2:**
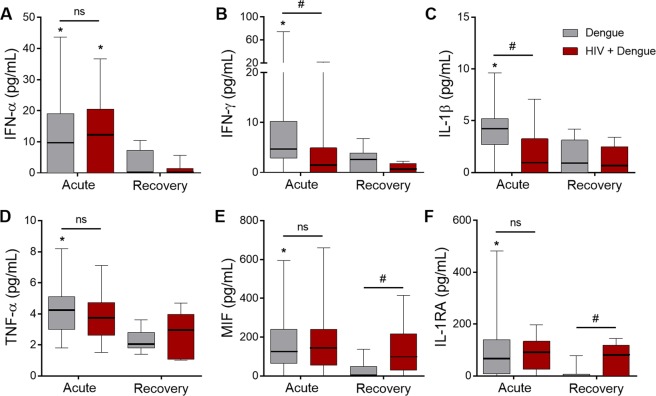
Circulating levels of inflammatory cytokines in patients with dengue infection or HIV plus dengue coinfection: The concentration of the cytokines IFN-α (**A**), IFN-γ (**B**), IL-1β (**C**), TNF-α (**D**), MIF (**E**) and IL-1RA (**F**) were quantified in plasma samples obtained at the acute or recovery phase of dengue illness from patients infected with dengue only or coinfected with HIV plus dengue (HIV + dengue). The horizontal lines in the box plots represent the median, the box edges represent the interquartile ranges and the whiskers indicate 5–95 percentile. *Signifies p < 0.05 compared to patients with the same profile of infection (dengue or HIV + dengue) at the recovery. ^#^Means p < 0.05 between dengue and HIV + dengue.

To gain insights into whether milder dengue illness in patients coinfected with HIV is a result of reduced DENV replication or increased viral clearance in consequence of chronic activation of antiviral immune response or the use of ART, we quantified the DENV genome in plasma samples from 25 PCR+ dengue infected patients (15 dengue infected and 10 HIV plus dengue coinfected). We found no difference in DENV viremia between the two groups (Fig. 1B), indicating that DENV replication and clearance were similar between patients with dengue regardless of HIV coinfection. In agreement, the plasma levels of IFN-α, a major mediator of antiviral immune response^48^, were also similar between patients infected with dengue and patients infected with HIV plus dengue (Fig. 2A).

### Dengue and HIV coinfection associates with lower levels of pro-inflammatory cytokines and platelet-derived chemokines

**Figure 3:**
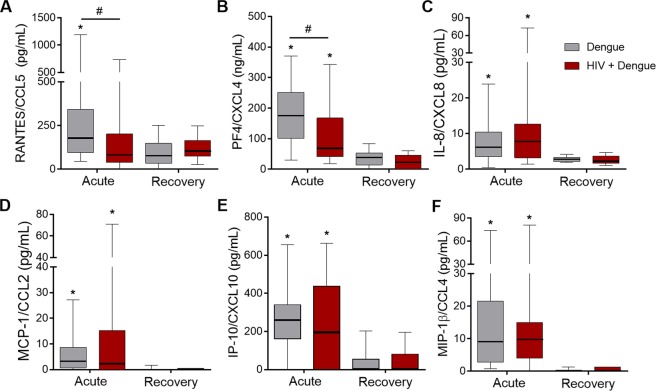
Circulating levels of chemokines in patients with dengue infection or HIV plus dengue coinfection: The concentration of the chemokines RANTES/CCL5 (**A**), PF4/CXCL4 (**B**), IL-8/CXCL8 (**C**), MCP-1/CCL2 (**D**), IP-10/CXCL10 (**E**) and MIP-1β/CCL4 (**F**) were quantified in plasma samples obtained at the acute or recovery phase of dengue illness from patients infected with dengue only or coinfected with HIV and dengue (HIV + dengue). The horizontal lines in the box plots represent the median, the box edges represent the interquartile ranges and the whiskers indicate 5–95 percentile. *Signifies p < 0.05 compared to patients with the same profile of infection (dengue or HIV + dengue) at the recovery. ^#^Means p < 0.05 between dengue and HIV + dengue infected patients.

Considering the major role of proinflammatory response in dengue pathogenesis, we hypothesized that the more benign clinical presentation in HIV plus dengue coinfected patients would be related to differences in inflammatory mediators present during dengue infection within the HIV background. We quantified the levels of cytokines (Fig. 2) and chemokines (Fig. 3) in plasma samples from the acute phase of dengue illness (40 dengue and 25 HIV plus dengue) compared with samples from the recovery (8 dengue and 7 HIV plus dengue). Similar to previous reports^18,49–51^, the pro-inflammatory cytokines IL-1β, IFN-γ, TNF-α and MIF were increased in plasma samples from patients with dengue at the acute phase compared to samples obtained after recovery (Fig. 2B–E). The anti-viral cytokine IFN-α and the anti-inflammatory cytokine IL-1RA were also increased in acute dengue infection (Fig. 2A and F). Importantly, plasma levels of the proinflammatory cytokines IFN-γ and IL-1β were reduced in HIV plus dengue coinfected patients compared to patients infected with DENV only (Fig. 2B,C), suggesting that continuing immune suppression or immune exhaustion in people living with HIV may affect the proinflammatory milieu during dengue infection. In patients coinfected with HIV, plasma levels of MIF and IL-1RA remained increased after recovering from dengue illness (Fig. 2E,F), which is consistent with persistent inflammation in HIV infected subjects under stable ART.

Plasma levels of the chemokines RANTES/CCL5, PF4/CXCL4, IL-8/CXCL8, MCP-1/CCL2, IP-10/CXCL10 and MIP-1β/CCL4 were also increased during the acute phase of DENV infection compared to the recovery (Fig. 3). Even though most of the chemokines were present at similar levels in samples from dengue infected and HIV plus dengue coinfected patients, the platelet-derived chemokines PF4/CXCL4 and RANTES/CCL5 were reduced in patients coinfected with HIV when compared to patients infected with DENV only (Fig. 3A,B). Of note, the chemokine PF4/CXCL4 is exclusively expressed by platelets and megakaryocytes, and its lower levels in plasma suggests defective platelet responses in HIV plus dengue coinfected patients.

### Thrombocytopenia and rates of platelet apoptosis are similar between dengue infected and HIV plus dengue coinfected patients

**Figure 4:**
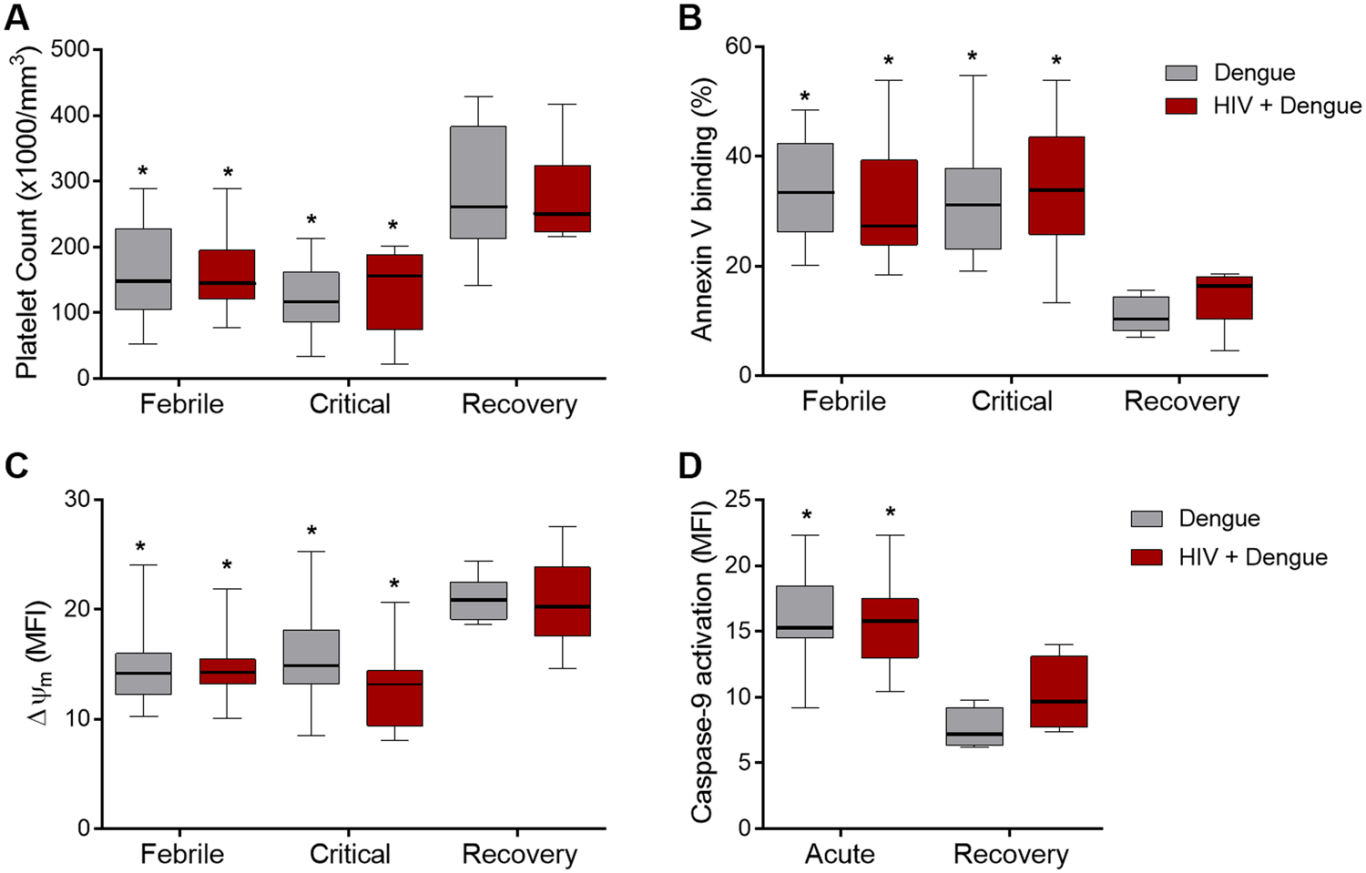
Thrombocytopenia and platelet apoptosis in patients with dengue infection or HIV plus dengue coinfection: (**A**) Prospective analysis of platelet counts at the febrile, defervescence and recovery phase of dengue illness in patients infected with dengue only or coinfected with HIV plus dengue (HIV + dengue); (**B**) The percentage of phosphatidylserine exposure measured by the binding of Annexin V on platelets from patients with dengue or HIV + dengue; (**C**) The fluorescence of the probe TMRE indicating the mitochondrial membrane potential (Δψ_m_) in platelets from patients with dengue or HIV + dengue; and (**D**) The fluorescence of the probe FLICA indicating active caspase-9 in platelets obtained at acute and recovery phase from patients with dengue or HIV + dengue. The horizontal lines in the box plots represent the median, the box edges represent the interquartile ranges and the whiskers indicate 5–95 percentile. *Signifies p < 0.05 compared to patients with the same profile of infection (dengue or HIV + dengue) at the recovery.

We then investigated whether lower levels of platelet-derived chemokines in HIV plus dengue coinfected patients result from lower platelet counts in HIV infected subjects. We prospectively followed the platelet counts in dengue infected and HIV plus dengue coinfected patients at the febrile and critical phases of dengue illness compared to the recovery. As shown in Fig. 4A, the drop of platelet counts reached similar levels in dengue infected and HIV plus dengue coinfected patients at febrile and critical phases of dengue illness. In agreement, the rates of platelet apoptosis, which is a key mechanism for platelet clearance in dengue^32,39,47,52^, were also similar between the two groups of patients as evidenced by increased phosphatidylserine exposure, collapse of mitochondrial membrane potential (ΔΨ_m_) and increased activation of caspase-9 (Fig. 4B–D). All HIV infected subjects followed until the convalescence phase recovered their platelet counts to normal reference values when recovering from dengue illness, which is consistent with absence of HIV-associated thrombocytopenia in the HIV infected individuals in our cohort.

### Platelets become activated during dengue infection regardless of HIV coinfection

**Figure 5:**
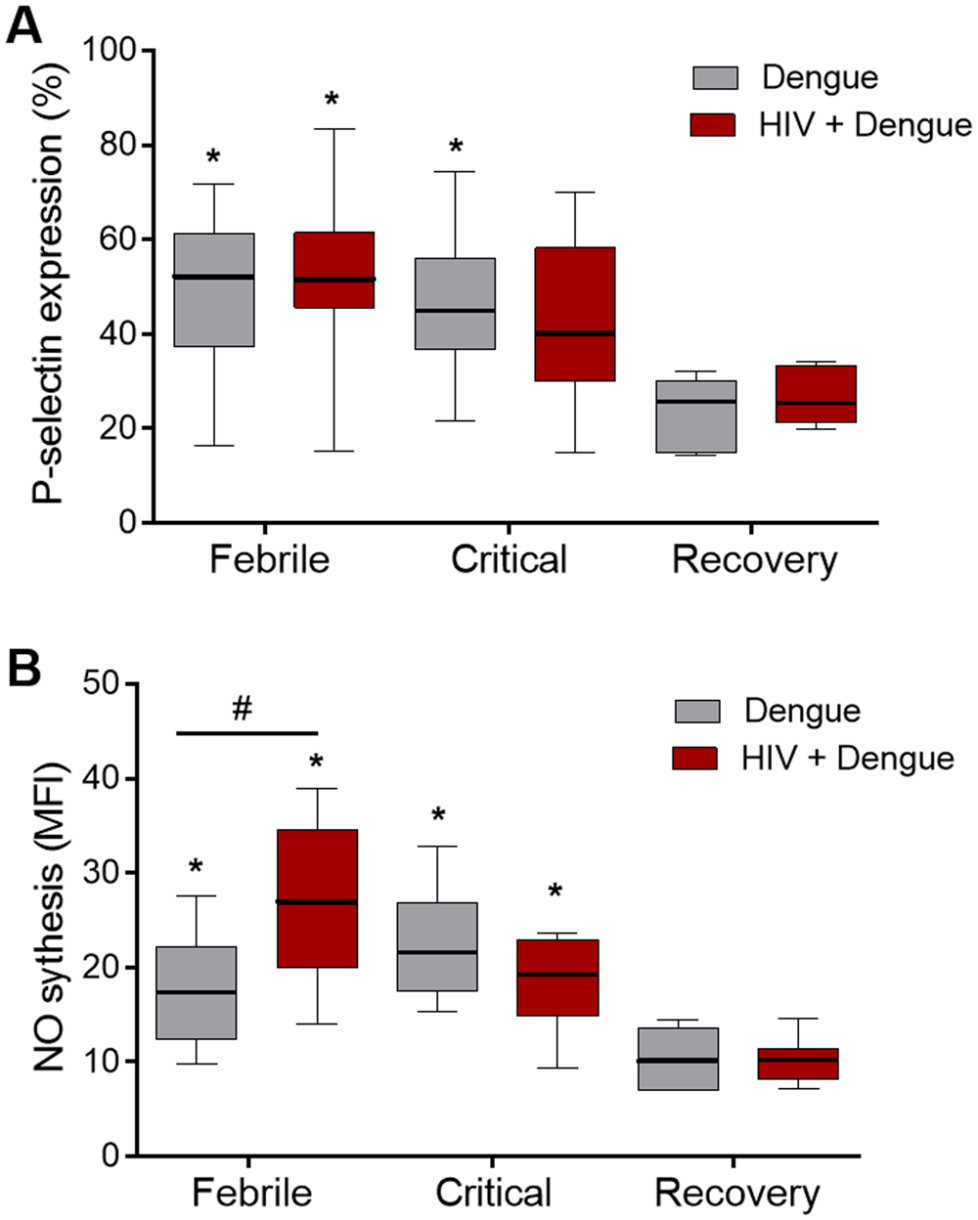
Platelet activation in patients with dengue infection or HIV plus dengue coinfection: Activation was assessed at the febrile, defervescence and recovery phases of dengue illness in freshly isolated platelets from patients with dengue only or coinfected with HIV and dengue (HIV + dengue). (**A**) The percentage of platelets with P-selectin (CD62P) surface expression in each condition; and (**B**) the fluorescence of the probe DAF-FM diacetate indicating NO synthesis by platelets. The horizontal lines in the box plots represent the median, the box edges represent the interquartile ranges and the whiskers indicate 5–95 percentile. *Signifies p < 0.05 compared to patients with the same profile of infection (dengue or HIV + dengue) at the recovery. ^#^Means p < 0.05 between dengue and HIV + dengue infected patients.

We and others have previously shown that platelets from dengue infected patients have features of cellular activation^33–35,39,47,53^. Platelet activation has been also reported in subjects chronically infected with HIV and there is evidence for persistent platelet activation even when virologic suppression is achieved through ART^38,41^. To assess platelet activation in patients coinfected with DENV and HIV-1 we evaluated the surface expression of the activation marker P-selectin (CD62-P) and the synthesis of nitric oxide (NO) in platelets obtained from dengue infected and HIV plus dengue coinfected patients at the febrile and critical phases of dengue illness compared to samples obtained at the recovery. Similar to previously reported observations^35,39,47,53–55^, platelet expression of CD62-P and synthesis of NO were increased at the febrile and critical phases of dengue illness (Fig. 5). Platelet activation and increased synthesis of NO were also observed in platelets from patients coinfected with dengue and HIV (Fig. 5). Even though coinfected patients presented lower levels of platelet-derived chemokines in circulation (Fig. 3), platelets became activated in coinfected patients at levels that were similar to patients infected with DENV only (Fig. 5A). Interestingly, the synthesis of NO by platelets was even higher in HIV plus dengue coinfected patients at the febrile phase when compared to platelets from dengue patients without HIV-1 infection (Fig. 5B). These results indicate that chronic HIV infection does not impair the ability of dengue illness to activate platelets *in vivo*.

### Platelets from HIV plus dengue coinfected patients secrete lower levels of RANTES/CCL5 and PF4/CXCL4 *ex vivo*

**Figure 6:**
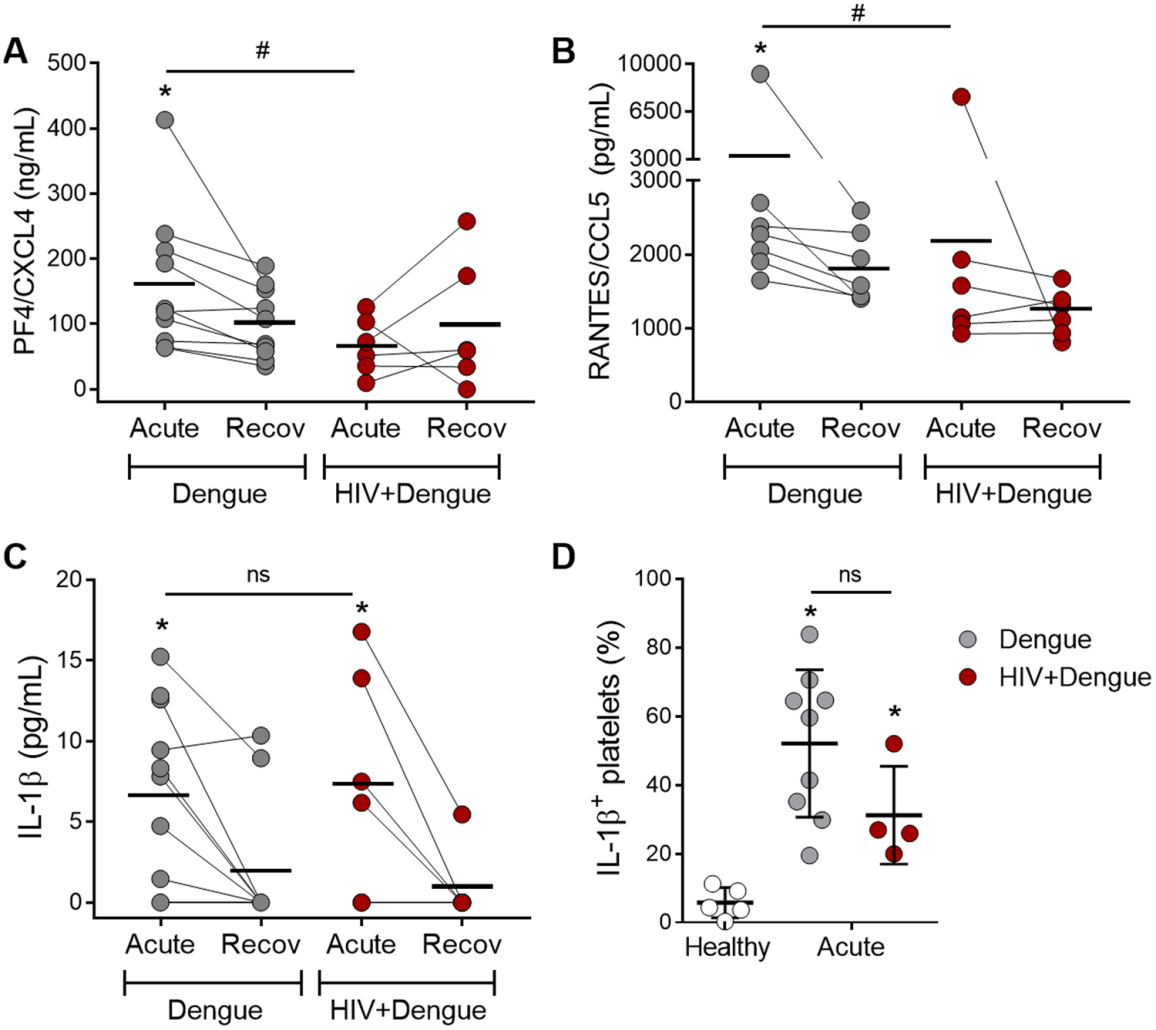
Secretion of stored chemokines and newly synthesized IL-1β by platelets from patients with dengue or patients coinfected with dengue and HIV: (**A**–**C**) Platelets (1 × 10^9^ per mL) obtained at the acute and the recovery phase of dengue illness from 10 patients with dengue and 6 patients with HIV plus dengue coinfection (HIV + dengue) were cultured *ex vivo* for 4 h. The concentrations of PF4/CXCL4 (A), RANTES/CCL5 (**B**) and IL-1β (**C**) in the supernatant of cultured platelets are shown. (**D**) Freshly isolated platelets from 9 dengue-infected patients, 4 HIV + dengue coinfected patients and 5 age- and sex- matched healthy volunteers were intracellularly labeled for IL-1β. The percentages of platelets expressing IL-1β are shown in each condition. Each dot represents values from one patient or healthy volunteer. The horizontal lines in each group represent the mean. The whiskers in panel D indicate the standard deviation. *Signifies p < 0.05 compared to patients at the recovery phase or to healthy volunteers. ^#^Means p < 0.05 between dengue and HIV + dengue infected patients. ns means nonsignificant.

We have recently reported that HIV-infected subjects under stable ART have evidence for persistent platelet activation and exhausted platelet α-granules content, even after virological suppression through ART^41^. We therefore hypothesized that exhausted granules in platelets from HIV-infected subjects before coinfection with DENV may affect platelet chemokine secretion during dengue illness resulting in lower levels of platelet-derived chemokines in plasma, as observed in Fig. 3. We then quantified the levels of the chomokines RANTES/CCL5 and PF4/CXCL4 secreted *ex vivo* by platelets isolated from dengue-infected or HIV plus dengue-coinfected patients. As shown in Fig. 6, platelets isolated in the acute phase from dengue-infected patients, but not platelets from HIV plus dengue-coinfected patients, released higher levels of PF4/CXCL4 and RANTES/CCL5 when incubated *ex vivo* compared to platelets from the same patients collected and examined at the recovery (Fig. 6A and B). In addition to granule-stored chemokines, activated platelets from patients with dengue also release newly synthesized IL-1β^33^, and IL-1β is also reduced in plasma from coinfected patients (Fig. 2C). In contrast to stored chemokines, platelets from HIV plus dengue coinfected patients secreted IL-1β at levels that were similar to patients infected with DENV only (Fig. 6C). Similarly, the percentage of platelets expressing IL-1β evaluated by flow cytometry in a smaller number of patients (9 dengue and 4 HIV + dengue) showed similar levels between dengue infection and HIV + dengue coinfection (Fig. 6D). These data indicate that platelet exhaustion of granule content may account for lower levels of platelet-derived chemokines in coinfected patients, while the secretion of newly synthesized IL-1β by platelets remains unaffected.

## Discussion

Despite virologic control accomplished by ART, people living with HIV still experience chronic inflammation and immune dysfunction, including persistent platelet activation and exhaustion^4,8–12,41,56,57^. Even though normal rates of CD4+ T cells and freedom from opportunistic infections are achieved, these immune changes potentially affect the outcome of endemic/epidemic infections that HIV-infected subjects are also exposed to. Here we show that dengue infection in people living with HIV under stable ART associates with milder clinical presentation characterized by reduced vascular instability and liver damage (Fig. 1 and Table 1). Consistently, dengue plus HIV coinfected patients also presented lower levels of plasma pro-inflammatory cytokines (IFN-γ and IL-1β) and platelet-derived chemokines (RANTES and PF4) when compared to patients infected with DENV only (Figs 2 and 3). Even though the degree of thrombocytopenia and the rates of activated platelets were similar between patients with dengue and patients coinfected with dengue and HIV (Figs 4 and 5), platelets isolated from coinfected patients secreted lower levels of stored-chemokines *ex vivo* when compared to platelets from dengue-infected patients (Fig. 6). Altogether, these results suggest that exhausted platelets from HIV-infected subjects release lower levels of granule-stored chemokines during acute dengue infection, this biologic feature when combined with reduced secretion of proinflammatory cytokines from other sources may contribute to lower risks of vascular instability observed in HIV plus dengue coinfected patients.

Coinfection with HIV has been previously shown to influence the outcome of endemic and epidemic viral infections including dengue and influenza A virus (IAV)^23,58^. For both viral infections, patients coinfected with HIV have been shown to present benign or worsened clinical syndromes depending on whether HIV is controlled by ART or not, respectively^23,58–60^. Similar to the present work, a previous study has shown that HIV infected subjects under stable ART presented a more benign clinical outcome during dengue illness^23^. In a small cohort primarily comprised by AIDS patients, however, DENV plus HIV coinfection was associated with increased risk of severe dengue^59^. Similarly, IAV infection in AIDS patients has been related to prolonged hospitalization, higher rates of mechanical ventilation and increased mortality^58^, while being under stable ART associated with benign course and increased survival^58,61–63^. Molecular analysis of the IAV mutation accumulation rates evidenced reduced IAV replication in HIV infected subjects, which may involve intrinsic anti-viral immunity by the restriction factor IFN-induced transmembrane protein 3 (IFITM3)^64^. In the present work, we did not observe any difference in DENV viremia and in plasma levels of type I IFN during dengue illness in HIV-infected subjects, suggesting that milder disease progression in HIV-infected subjects coinfected with DENV or IAV may involve differential mechanisms.

Even though the pathophysiologic mechanisms underlying severe dengue illness are not completely understood, it is widely accepted that unbalanced release of inflammatory cytokines and chemokines plays a major role in disease pathogenesis^18–22^. For instance, cytokines that signal antiviral immune response as type I IFN and chemokines responsible for the recruitment of cytotoxic immune cells as MIP-1β/CCL4 have been associated with benign clinical evolution and mild dengue^18,65,66^. However, the levels of IFN-α and MIP-1β were not associated with milder dengue illness in HIV-coinfected patients in the present study. Circulating levels of the proinflammatory cytokines IFN-γ and IL-1β and the vasoactive chemokines RANTES/CCL5 and PF4/CXCL4, on the other hand, were reduced in coinfected patients when compared to dengue infection alone. While people living with HIV present chronic inflammation and increased levels of proinflammatory cytokines compared to uninfected subjects^10,11,67,68^, people living with HIV under stable ART presented reduced levels of proinflammatory cytokines and chemokines during acute DENV infection compared to patients infected with DENV only. These data suggest that continuing immune dysregulation in people living with HIV may affect the proinflammatory response during dengue illness.

Our group and others have previously shown that increased platelet activation participates in immune and inflammatory response in dengue illness^33–35,39,47,53^. Activated platelets in dengue patients contribute to inflammatory amplification by a variety of mechanisms, including the release of stored and newly synthesized mediators and by interacting with leukocytes through P-selectin-mediated adhesion^33–35^. Among stored factors, platelets from dengue-infected patients and platelets infected with DENV *in vitro* have been shown to secrete higher levels of the co-stimulatory molecule sCD40L and the chemokines PF4 and RANTES^35,69^. Activated platelets from patients with dengue and *in vitro* infected platelets have been also shown to synthesize and secrete IL-1β^33^. In the present work, platelet responses were primarily similar between dengue infected and HIV plus dengue coinfected patients. However, when platelets isolated from patients were incubated *ex vivo*, platelets from coinfected patients secreted lower levels of RANTES/CCL5 and PF4/CXCL4, but not IL-1β, compared to patients with dengue without HIV coinfection. These data suggest that platelets from patients coinfected with dengue and HIV present a defect specifically in the secretion of granule-stored factors, which is consistent with exhaustion of granule content as previously reported in people living with HIV^41,70^.

It has been shown that platelets from HIV-infected subjects or AIDS patients secrete lower levels of RANTES when stimulated with pro-coagulant agonists *ex vivo*^41,70^. Even though RANTES secretion by platelets from AIDS patients reached near normal levels after 12 weeks on ART, it remained defective compared to healthy volunteers^70^. Consistently, we have recently shown in a cohort of HIV-infected subjects under stable ART that platelets still demonstrate features of exhaustion including reduced labeling for α-granules and deficient secretion of stored chemokines under thrombin stimulation^41^. Platelet exhaustion may occur as consequence of chronic platelet activation. Platelets from AIDS patients present features of increased activation that decrease after ART initiation^70–74^, but there is strong evidence that platelet activation in HIV infected subjects persists after months to years of virologic suppression by ART^41,75–78^. Here we show that exhausted platelets in people living with HIV under stable ART secrete lower levels of granule-stored chemokines during coinfection with DENV, which impacts the levels of these chemokines in circulation.

The specific cell sources of the cytokines and chemokines that were changed in dengue by coinfection with HIV remain elusive. While lower plasma levels of PF4/CXCL4 in coinfected patients can be explained almost exclusively by platelet degranulation fatigue, reduced levels of RANTES/CCL5, IL-1β and IFN-γ may be influenced by suppression or dysfunction of many components of the immune system, including T cells, which are main sources of IFN-γ. It has been shown that even when virological control and normal rates of CD4+ T cells are achieved through ART, exhaustion of T cell effector responses persists in people living with HIV^56,79,80^. It is important to note that CD4+ and CD8+ T cells are chief mediators of both pathogenesis and viral control in dengue^81–84^. Interestingly, patients coinfected with dengue and HIV presented increased expansion of CD8+ related to CD4+ T cells and increased activation of both T cell subsets compared to dengue patients without HIV coinfection^23^. Of note, IL-1β and IFN-γ are major drivers of vascular inflammation and endothelial dysfunction, and have been both associated with increased vascular permeability and the onset of shock in severe dengue^18,85^. Together with chemokines, lower levels of these cytokines may be involved in milder dengue syndrome in people living with HIV.

Finally, we cannot exclude a possible effect of different ART regimens on platelets or other immune cells and consequent influence on the outcome of dengue. Unfortunately, because of the multiplicity of ART regimens and the limited sample size in our cohort of coinfected patients we could not address the influence of this issue in the current study. In previous studies, abacavir-containing ART has been associated with platelet hyperreactivity while the integrase inhibitor raltegravir was associated with reduced platelet activation^76,86,87^. In a recent clinical study, however, switching from integrase-inhibitor naïve therapy to raltegravir-based regimen did not attenuate platelet activation^88^. Importantly, only 2 patients (6.7%) in our cohort were taking abacavir at the time of inclusion. In *in vitro* experiments, stimulation with abacavir or its metabolite carbovir-triphosphate has been shown to potentiate platelet degranulation and aggregation in response to prothrombotic agonists^89,90^. Mechanistically, stimulation with abacavir or carbovir-triphosphate has been shown to competitively inhibit the activity of guanylyl cyclase when compared to non-guanosine nucleotide analogues, preventing the inhibitory effect of NO, a major negative-regulator of platelet activation^89,90^. Interestingly, we observed increased NO synthesis in platelets from HIV + dengue coinfected patients when compared to patients infected with DENV only. Increased production of NO has been reported in platelets from patients with dengue alongside lower platelet aggregation^54^. Nevertheless, new studies are still necessary to determine causal relationship between increased NO synthesis and platelet disfunction in dengue-infected or HIV + dengue coinfected patients.

In summary, we report that dengue infection in people living with HIV under stable ART is associated with more benign clinical presentation and lower risk of vascular instability. Our data suggest that HIV-driven reprogramming of host homeostasis alters the regulation of the immune response and, consequently, the inflammatory milieu during dengue infection, with important consequences to disease progression and severity. Platelets from HIV infected subjects, although still capable of increased activation during dengue illness, participate in this adapted immune response by secreting lower levels of stored chemokines. This report contributes to a better understanding of the events underlying immune reprograming during dengue coinfection in people living with HIV, and may be relevant to other coinfections that may involve similar immune mechanisms, especially regarding platelet-mediated immune responses.
